# An investigation of the efficacy and mechanism of contrast-enhanced X-ray Computed Tomography utilizing iodine for large specimens through experimental and simulation approaches

**DOI:** 10.1186/s12899-015-0019-3

**Published:** 2015-12-21

**Authors:** Zhiheng Li, Julia A. Clarke, Richard A. Ketcham, Matthew W. Colbert, Fei Yan

**Affiliations:** Department of Geological Sciences, University of Texas at Austin, Austin, TX USA; Department of Civil and Environmental Engineering, Rice University, Houston, TX USA

**Keywords:** I_2_KI, Formalin, Staining, Diffusion, Sorption, CT-scan, Soft-tissue contrast, Aves

## Abstract

**Background:**

Iodine-based solutions have long been known to be effective in aiding the differentiation among soft tissues in both fundamental anatomical research and for clinical diagnoses. Recently the combination of this particular contrasting agent with micro-computed tomography (micro-CT) has resulted in an array of high-quality image data, in which anatomical structures not visible in conventional CT can be identified and quantified. However, there has been only limited data available to inform detailed protocols for staining large specimens. Further, modeling of the staining mechanism has focused on simple diffusion processes.

**Results:**

A low concentration of iodine-based buffered formalin solution with a long staining period was used to visualize soft-tissue structures in a large goose head. The staining effect was analyzed by serially measuring the micro-CT profiles across coronal sections throughout the staining period. Regular replacement of the staining solution combined with a longer staining period significantly improved contrast within tissues. A simplified one-dimensional Diffusion-Sorption model with a three-zone domain was used to simulate the diffusion process by calculating the concentration profile of iodine across the adductor region, which fits well with the experiment data. Observations of changes in the concentration of the staining agent and simulation results suggest that the sorption of iodine by tissues significantly affects the effective diffusion coefficient for the contrasting agent.

**Conclusions:**

The Diffusion-Sorption model better explains previously reported difficulties in staining large samples comprised of tissues with high partition coefficients (*K*_*d*_). Differences in partition coefficient (*K*_*d*_), bulk density (*ρ*_*b*_), and porosity (*θ*) could further explain the observed variation in staining rate and maximal staining effect among different tissues. Recommended protocols for staining large specimens are detailed.

## Background

In the past five years, extensive progress has been made in the application of iodine solutions to increase radiographic contrast in soft tissues for micro-X-ray computed tomography (micro-CT) scans of small organic samples [1–4]. Iodine, the most widely-used contrasting agent in micro-CT scans, has several obvious advantages, such as low cost, perceived non-destructiveness, low toxicity, and rapid staining [1, 2, 5, 6]. Datasets with resolution comparable to histological approaches have been obtained from diverse samples including the whole body of mice, neonatal alligator heads, rat hearts, small insects, avian embryos, and even small flowers [1, 2, 7–9]. Based on CT imaging of these stained specimens, accurate and detailed three-dimensional (3D) structures were reconstructed and assessed through comparison with histological data [9, 10]. Successful trials of this contrast-enhancing technique in micro-CT imaging have shown the importance of three conditions: (1) the effective and efficient penetration of iodine through organic tissues, (2) different X-ray attenuation for different tissues after the staining, and (3) the high resolution of CT images that derived from micro-CT scans (1–50 μm resolution) [6, 11]. The concentration of iodine solution used varied from 1 % to 25 % and the duration of staining ranged from a few hours to a few weeks, even though most samples are generally less than 1 cm in diameter [1, 2, 7–9]. Generally, higher concentrations of iodine yielded higher rates of iodine penetration but also cause higher degrees of tissue shrinkage [6, 12].

As noted, previous successful applications of iodine contrast-enhanced CT have been achieved for small specimens such as various viscera, mammalian and avian embryos, and small fishes. Standard protocols for effective staining of these small specimens have been documented and validated by an array of different laboratories [1–10]. Unfortunately, application of this approach to even slightly larger samples (still quite small, e.g., ~ 2–4 cm in diameter) has not been reported to be equally successful [5, 9]. Few experiments have been undertaken using these larger specimens, in part because of concerns about the duration of the staining process given proposed size limitations for the passive diffusion capacity of iodine molecules [5]. Efficient and effective penetration of larger specimens even when using solution with the same, or even higher iodine concentrations has proven largely elusive for samples larger than 5 cm in diameter [5, 13]. Size has placed another constraint on imaging these samples; they cannot be accommodated in most micro-CT scanners. These two factors have limited the application of contrast-enhanced CT for samples over 5 cm. As a result, effective staining regimes and protocols for larger specimens have not been defined.

The proposed physical chemistry underlying the staining mechanism and in particular the interaction of iodine with tissues has only been generally outlined [6, 15]. The proposed process of sorption or adsorption refers to the binding of iodine species onto molecules of glycogen, lipids, or other carbohydrates that comprise the tissues [8, 14, 16]. However, no modeling to date has considered how the sorption reaction may affect the diffusion rate of iodine throughout the staining period. Similarly, only a simplified diffusion model has been proposed to explain concentration changes in iodine at a certain position within small embryos [6, 15]. The potential influence of the reaction of iodine with distinct tissues on the staining process over longer staining periods has not been discussed or quantitatively analyzed. In addition, the pattern and timing of the exchange of iodine in the solution and within different tissues is also poorly understood. Only simple diffusion processes driven largely by differences in solute concentration have been discussed [15]. However, both the diffusion and sorption behavior of iodine within different tissue types must affect the selection of staining parameters (e.g., concentration, duration, and temperature) to maximize contrast for specific studies. An improved understanding of both is essential for describing the staining mechanism and developing effective protocols, especially for larger specimens with longer staining periods.

Here we investigate staining protocols for large specimens (~4 cm in diameter; volume ~125 mL) using a modified iodine-based buffered formalin as the staining solution. We explore longer staining durations (up to 49 days) than have been previously considered [1, 2, 5, 9]. CT scans are serially taken throughout the staining period to monitor changes in contrast in distinct tissue types. We also consider the effectiveness of regular replacement of the low-concentration iodine solution for increasing staining while minimizing tissue shrinkage. Potential explanations of the experimental results are then explored in simulations. These simulations use a Diffusion-Sorption (D-S) model [17, 18] that takes into account transport of iodine within tissues via diffusion as well as the effect of iodine-tissue interactions (sorption). Different characteristics of tissues are defined to simulate the diffusion process more accurately compared to previous simple diffusion model [15].

## Results

Over the 49-day staining period, different patterns of change in tissue contrast were observed in earlier and later stages of staining and after the input of additional iodine. The first round of immersion in the contrasting agent primarily resulted in staining of exterior regions directly in contact with the solution. The latter two rounds of staining, after the iodine solution had been replaced resulted primarily in changes in CT values in more internal regions. The Diffusion-Sorption (D-S) model was found to effectively describe the pattern of contrast increase across different tissues over time as shown by fitting the experimental data in the adductor region.

### Staining observed in tissues following the first round of immersion (scans A and B) in the iodine solution

#### Skin and integumentary structures

**Fig. 1:**
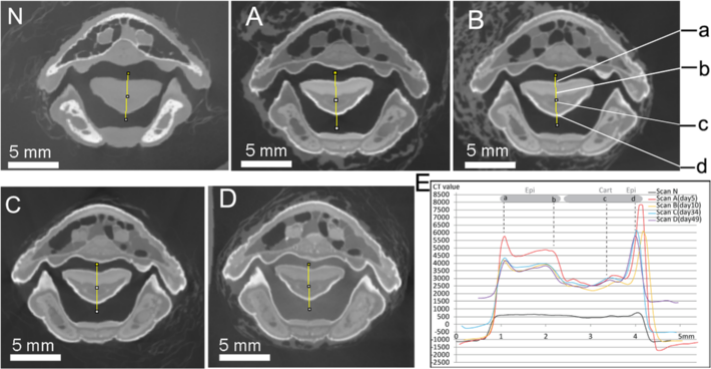
The coronal section of CT images crossing the beak and cranial region of the tongue (**n**, **a**-**d**) with CT value plots for targeted tissues selected (**e**). **n** is the control scan, and **a**, **b**, **c**, and **d** correspond to scans after 5, 10, 34, and 49 days of staining, respectively. The straight yellow line in N-D represents the position (**e**) where CT values were measured (using ImageJ v1.48). The changes in CT values were used for tissue identification, correlation among scans and their referenced positions on the straight line (*a*-*d*). Line colors in E correspond to CT scans as: black-N, red-A, yellow-B, blue-C, and purple-D. Anatomical abbreviations: Cart, cartilage; Epi, epidermis

**Fig. 2:**
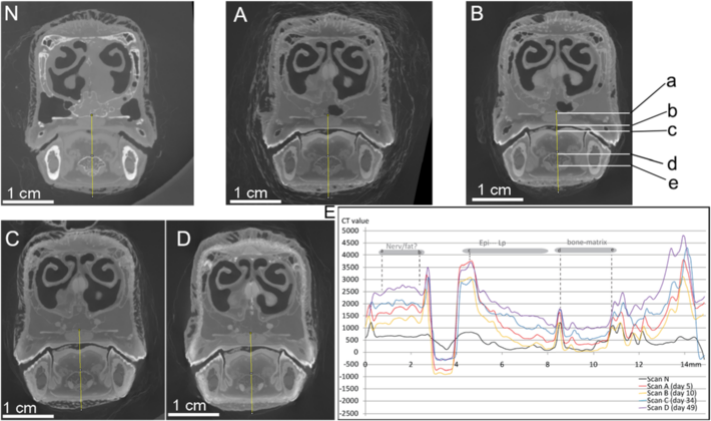
The coronal section of CT images crossing the nasal capsule and the middle region of the tongue (**n**, **a**-**d**) with CT values plots for targeted tissues selected (E). **n**, **a**, **b**, **c**, and **d** correspond to the five scans described as in Fig. 1; the same color regime was used for the CT plots as well. Distinct CT values with tissue identification were labeled and correlated between the plots (**e**) and positions on the line (**b**: *a*-*e*). Anatomical abbreviations: Epi, epidermis; Lp, lamina propria; Nerv/fat?, nerve or fat tissues

**Fig. 3:**
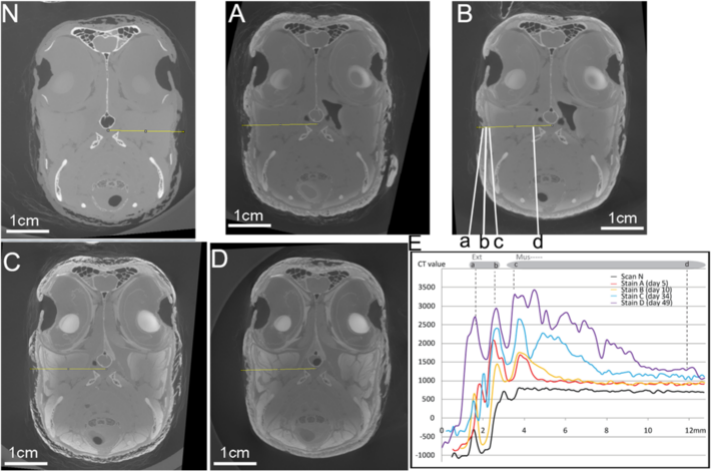
The coronal section of CT images crossing the eye and adductor chamber (**n**, **a**-**d**) with CT plots for the adductor region (**e**). **n**, **a**, **b**, **c**, and **d** correspond the respectively same scan as in Figs. 1 and 2; CT plots indicate an overall decreasing trend from the exterior margin of the muscle to more interior side (see detail in text). Exterior region and interior muscles were well identified and correlated with CT images and the plots (**b, e**: *a-d*). The same color regime was used for the CT plots as in Figs. 1 and 2. Anatomical abbreviations: Ext, external zone; Mus, muscles

**Fig. 4:**
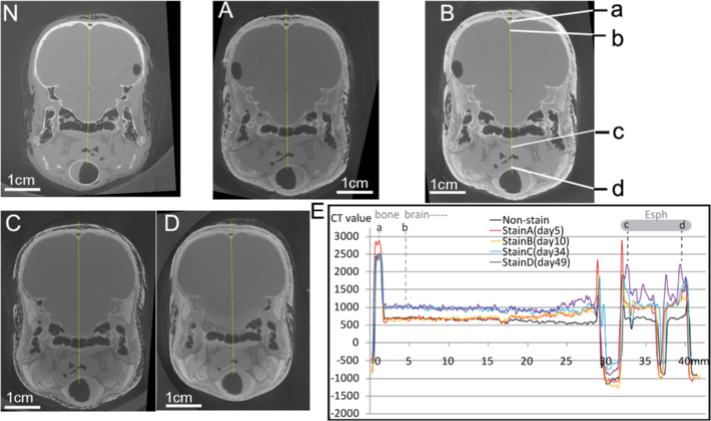
The coronal section of CT images crossing brain, esophagus, and trachea (**n**, **a**-**d**) with CT values plots for selected regions (*a*: bone, *b*: brain, and *c-d*: esophagus). **n**, **a**, **b**, **c**, and **d** correspond to the five scans as described in Figs. 1, 2 and 3. The identified CT values in E, F show similar CT values for the bone and brain (*a*-*b*), but clearly different values for the esophagus (*c*-*d*) over the staining. The same color regime was adopted for the CT plots. Anatomical abbreviations: Esph, esophagus

Early in the staining process (days 1–10), the uptake of iodine occurred primarily in the skin, integumentary structures, and oral and tongue epithelium, as seen in the change of CT values in these regions (Figs. 1-a, b, e and 2-a, b, e). Muscles, bone, and brain tissues did not show major changes in CT values (Figs. 3-a, b, e and 4-a, b, e). Integumentary structures such as feathers do not stay in fixed positions between individual scans and commonly rotate around their follicles, making the identification and comparison of exactly the same tissue in these feathers from scan to scan difficult (Fig. 3e: *a-b*). As a result, only the peak value and the range of CT values were compared for feathers and skin among scans A and B.

**Fig. 5:**
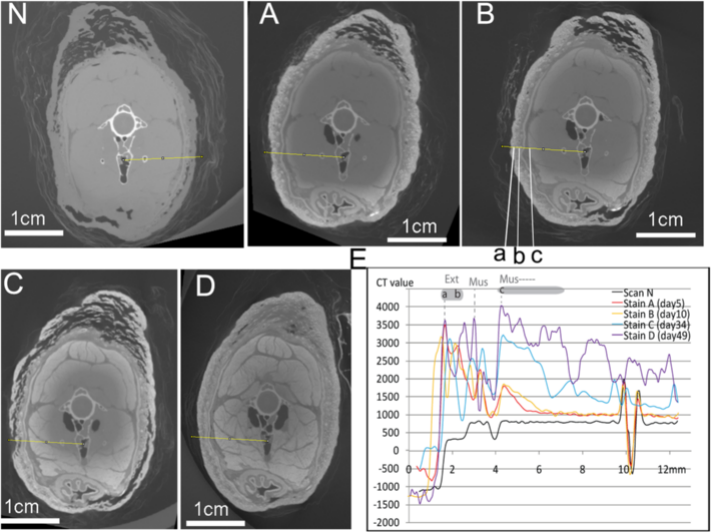
The coronal section of CT images crossing the neck (**n**, **a**-**d**) with CT values plots (**e**). The overall increased enhancement of staining effect is clearly indicated by both CT images and the CT value plots over staining. **n**, **a**, **b**, **c**, and **d** correspond to the same scan as described in Figs. 1, 2, 3 and 4 and the same color regime was used for CT plots. Anatomical abbreviations: Ext, external zone; Mus, muscles

As expected, feathers and the skin were the first tissues stained, as they lie in direct contact with the solution containing the contrasting agent. The CT values for feather and skin were in the 1000–2000 HU range, much higher than values measured in most other interior tissues in scans A and B (Figs. 3-a, b, e and 5-a, b, e).

#### Epithelium and connective tissue

The staining effect varied significantly among epithelia in different regions; for instance, the epithelia in the oral cavity and the lingual region were much better stained than epithelia in the esophagus in both A and B-scans. Higher CT values were observed for epithelia more exposed to the staining solution. In comparison with connective tissue, epithelia showed higher CT values in scans A and B.

Epithelia and connective tissue showed different CT values (Figs. 1-a, b and 2-a, b) in scans A and B. For the cranial portion of the tongue (Fig. 1-a, b), the CT value of the dorsal epithelium was around 3500–5500 HU (Fig. 1e: Epi); this value was higher than that of the cartilage deeper within the specimen (around 2500–3000 HU) and also higher than some of the observed values in the skin and feathers. The ventral epithelium has even higher CT values than the dorsal epithelium, with a peak value of over 5500 HU (Fig. 1e: *a*). CT values for the lingual epithelia were higher than that measured for the epidermis described above in scans A and B.

In the caudal portion of the tongue, similarly, the highly stained dorsal epithelium was distinguished from ventrally adjacent connective tissues (Fig. 2e: Lp and Cart). CT values for the superficial, or dorsal, epithelium were about 3000–3500 HU while they were only 1500–2000 HU for the adjacent connective tissues. Only minor variation in CT values was discerned between the exterior and interior region of the lingual epithelia in scans A and scan B (Figs. 1, 2). In contrast, a gradual decrease in CT values toward the middle sections of connective tissues was observed. CT values of connective tissues in scans A and B were lower than those in the skin and epithelium even for those close to the staining solution. For instance, the thin superficial fasciae between the skin and major jaw muscles (Fig. 3e: between *b* and *c*), yielded low CT values of about 1000 HU.

#### Muscle

For muscles in scans A and B, the peak CT values for the stained portions varied in different regions of the head (Figs. 3-a, b and 5-a, b). The stained muscles were generally only within a few millimeters (~2 mm), right beneath the skin, with a peak value of about 1500–1750 HU (Figs. 3e, 5e). Along the diffusion direction, the CT values for muscles fell gradually through the stained region to a transitional region (Figs. 3e, 5e). However, CT values for the interior muscle tissues within the treated samples were generally around 1000 HU; this number is still higher than that of the same tissues in the un-stained “control” scan (N, i.e., around 750 HU). The profile of CT values was quite similar between the muscles in the adductor region and those in the cervical region with respect to peak values (Figs. 3e, 5e: Mus) in scans A and B.

#### Bones and brain

No significant changes in CT values due to staining were detected in the bone and cerebrum, and the shrinkage (<1 %) of these tissues also was negligible in scans A and B (Fig. 4-n, a, b).

### The pattern of staining observed in distinct tissues following the addition of new iodine solution (scan C and D)

Between scans B and C, the solution containing the concentrating agent (600 mL 3 g [I_2_ + KI]/100 mL buffered formalin solution, i.e., 2.97 % w/v I_2_KI) was fully replaced (day 24; see Methods). A second solution replacement (600 mL, 2.97 % w/v, I_2_KI) was implemented on day 38, 11 days before scan D.

#### Skin and integumentary structures

The CT values of epidermis and skin continued to increase in scans C and D in the adductor region. The peak values for these tissues increased from 2000 HU to over 3000 HU (Fig. 3). For feathers and skin in the cervical region, that had already reached a high CT value (3000–3500 HU) during earlier scans (A and B), the peak value did not significantly change in later scans C and D (Fig. 5e: *a*).

#### Epithelium and connective tissue

For epithelia in direct contact with the staining solution, CT values increased slightly (2500–3500 HU; Fig. 2e). For the more interior epithelia, however, the CT values continued to increase, as shown in the esophageal region (1000 to 1500 HU; Fig. 4e). Although composed of similar tissues, the esophagus had much lower CT values in comparison with that of the lingual epithelium. Located near the center of the specimen (Fig. 4), most of the esophagus had CT values only around 1500 (HU) in scans C and D, lower than the epithelium in the tongue (mostly over 2000 HU). In addition, the external wall (muscularis externa) and internal layer (squamous epithelium) of the esophagus showed higher CT values than the tissues in between (Fig. 4e).

For the connective tissues underlying the epithelia (e.g., lamina propria), a limited increase was discerned, from around 1500 HU in earlier scans (A and B) to around 1500–2000 HU in the later scans (C and D). In general, the connective tissues (lamina propria, fasciae, and cartilages) were distinguished from other nearby tissues (e.g., muscle and epithelium) by showing relatively low CT values. For instance, superficial fascia can be distinguished from the skin and the muscles, similarly for the deep fascia that existed within the muscular fibers (Figs. 3, 5). These connective tissues were clearly lower in CT contrast than the muscles nearby, in which CT values were largely increased.

A small increase in cartilage CT values was discerned, similar to that seen in other connective tissues (e.g., lamina propria). The maximum CT value for cartilage was less than 2500 HU in later scans (C and D; Fig. 1), lower than the stained muscle tissues and the epithelia. The CT values of lamina propria and bony matrix fell in a similar range, and it was difficult to distinguish them based only on CT values (Fig. 2).

#### Muscles

For scans C and D, the CT values of muscles almost doubled in comparison to those in scans A and B. There was a dramatic change in staining effect in both the more exterior and interior regions of muscles, in comparison with the increases observed in other tissues discussed above. For more exterior muscle regions, which had been already stained, the peak values continued to increase (2500–3500 HU); the value even exceeded the peak value of the integument next to the muscle (Figs. 3e, 5e: *a*-*b*).

#### Bones and brain

For cranial elements as well as brain tissues housed inside of the cranial cavity, increased staining duration and the addition of new solution did not produce differences in observed CT values. We observed similar CT values across scans A to D when compared to values in the control scan (Fig. 4e: *a*-*b*) for both bone (2500–3000 HU) and brain tissues (500–1000 HU). This pattern indicates that the sorption of iodine by bony tissue was negligible over the whole staining process. Furthermore, the brain tissues did not show evidence of being stained in contrast to both the neonatal and embryo specimens previously reported [9, 15]. These results suggest that well-mineralized bone is a serious obstacle to the diffusion of triiodide (herein, I_3_^−^) which may explain why the brain tissues were stained in both the neonatal and embryonic specimens [9, 15] but not here in this adult specimen. However, it should also be noted that those previous studies on neonates and embryos used very different protocols with more than triple the iodine concentration (e.g., 11.25 % Lugol's iodine) and much shorter staining durations (1–4 weeks). They observed variable tissue shrinkages in the processed specimens, with enhanced shrinkage occurring in isolated tissues without skeletal support [12].

### Simulations

**Fig. 6:**
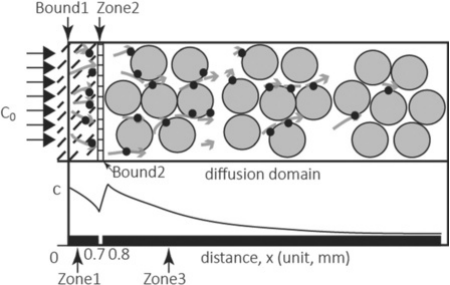
The Diffusion-Sorption model used for simulation of the experiment. Bound 1 indicates the start of iodine diffusion and Bound 2 defines the end of zone 2 (connective tissue) as well as the start of zone 3 (muscular tissues). Zone 1, Zone 2 and Zone 3 comprised the diffusion domains under study. C_0_ is the initial concentration of the staining solution and the curved line indicates hypothetical concentration changes in the diffusion domains. Figure legend: dash lines-feathers; large circles-muscle cells; the solid small dots-sorption of iodine by tissues

A simple diffusion model has been previously used to attempt to explain changes in iodine concentrations at a certain position in a mouse embryo given different staining durations [15]. However, our experimental data show that the staining effect varies significantly by tissue type, which indicates that the effective diffusion coefficient is not uniform for different tissues. In addition, there was a significant and continuous increase in the CT values at the edge of the skin and muscles during the staining, even though the solution concentration had not been raised. The concentration at this boundary should be determined by the initial concentration (Fig. 6: C_0_) based on the assumptions underlying the previously proposed diffusion model, the violation of which made its use problematic for explaining the iodine staining process. The variation in the staining effect observed in different tissues and the pattern of radiographic contrast increase through the diffusion domains cannot be explained by the simple standard diffusion model. Therefore, two improvements were implemented in a Diffusion-Sorption (D-S) model: (1) different tissues types were defined: skin, connective tissue, and muscles, and (2) different sorption (or adsorption) partition coefficients (*K*_*d*_) for iodine by the three tissues were taken into account. Sorption, as used here, refers to any attachment or binding of iodine with molecular components (e.g., glycogen and lipids) of tissues.

**Fig. 7:**
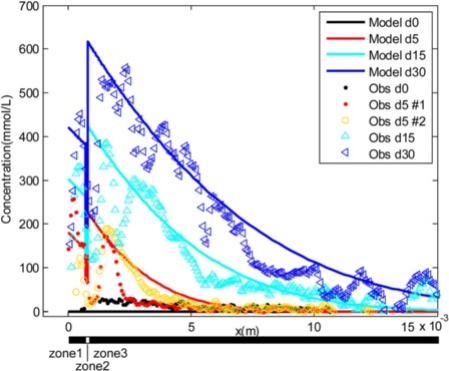
The similar pattern obtained from both modeling and experimental data to show the temporal and spatial profile of iodine concentration in the selected line (Fig. 3e: *b*-*d*). Model d0, d5, d15, d30 correspond with scans N, (A/B), C, and D respectively. Obs d0, d5#1, d5#2, d15, and d30 are the concentrations of iodine estimated through measurement of CT values in scans of N, A, B, C, and D. Note: because not much staining effect increase occurred between scan A and scan B, and no additional iodine was added to the system, the staining period between scan A (day 5) and the day the solution was first replaced (day 24), was treated as an ineffective staining interval. Therefore, scans A and B were treated as equivalent to day 5 in the modeling scenario. This ineffective staining interval was subtracted from the actual staining duration in the modeling for scans C and D (15 days and 30 days). By this recalibration of staining duration, a constant flux at the boundary condition in the model is generally expected to be met by maintaining the solution concentration in a certain level. Zone 1, Zone 2 and Zone 3 were labeled along the X axis

The CT values measured across the adductor region (Fig. 3) in these scans (N, A, B, C, D) were used to define the equivalent concentration of iodine within the tissues (see Methods for the conversion). The transformed concentration profiles were then used for the evaluation of the dynamics of iodine movement through the specimen over different staining durations by the implementation and assessment of the fit of the D-S numerical modeling results (Figs. 6, and 7). Four model parameters (*K*_*d*_ for three tissues and *N*_*bc*_) were determined through a trial-and-error process that adjusted the parameter values to match the model to experimental data (Figs. 6, and 7; Table 3). *K*_*d*_, the partition coefficient, describes the sorption of iodine between solid phase (tissue) and solute phase (i.e. *K*_*d*_ = *c*_*solid*_/*c*). The boundary flux (*N*_*bc*_) defines the boundary condition for the diffusion model, which is a parameter affected by the concentration of staining solution used.

The final estimated value for *K*_*d*_ in skin, connective tissues and muscles were 200 L/kg, 100 L/kg, and 200 L/kg respectively. The estimated *N*_*bc*_ was 1.4*10^−6^ m*mmol/(L*s). The porosity (*θ*) for tissues, although set as a constant (0.8) in our model, is also likely to have influenced the staining effect. As shown in Fig. 7, the model quite accurately predicts both the spatial and temporal profiles of iodine concentration in the target regions, including higher iodine concentrations in the skin and muscles, but very low concentrations in the connective tissue. The early peak concentration in the skin and muscular tissues is precisely captured by the model (Fig. 6: Bound 1 and 2; Fig. 7). The detailed pattern of changes in estimated iodine concentrations within muscles, skin or other tissues across a staining period has not been described or approached via modeling or simulations in previous studies [6, 15].

## Discussion

### Staining inefficiency and difficulty for different tissues

Comparison of scan B (day 10) to scan A (day 5) across various coronal sections reveals only limited changes, as shown in both CT images and CT values measured (Figs. 1, 2, 3, 4 and 5). Although there were still iodine molecules in the solution on day 5, the staining effect appeared to be stagnant in the latter stages of the first-round staining (day 5-day 10), and also for the rest of first-round staining (~ day 23). A similar result was reported by other investigators [6]. Here, we refer to this period as a “stagnant interval”, caused by deficient iodine in the staining solution. The sorption of iodine by the epidermis, dermis and exterior muscles led to iodine concentrations higher than those of the staining solution in these exterior regions, which could severely inhibit further diffusion. This result was consistent with a continuous accumulation or binding of the iodine with the muscular cells, specifically with the glycogen and lipids [14, 16].

Based on the experimental results, the pattern in iodine sorption was observed to be markedly different among tissue types (e.g., connective tissue, muscles, and skin). Larger muscles required a longer staining duration and a change of staining solution to stain completely. By contrast, the staining of epithelium, feathers, and skin was a comparatively fast process, in which they show a faster rate of reaching high iodine sorption and resultant high CT values. Therefore, their peak CT value did not significantly change in later scans C and D, compared to the earlier scans (Figs. 1, 3, 5). For muscle tissues, the major increase of CT values mostly occurred in the late staining stage as seen in scans C and D, especially for the interior muscular tissues. This means the sorption of iodine by muscles is a comparatively slow process, especially for the large bulky muscles (e.g., the jaw muscle complex, the cervical muscles). Therefore, a longer staining duration is required and justified here. By contrast, the increase of CT values in connective tissue (i.e., fasciae) with longer staining duration was limited (see scans C and D). This indicates that their low capacity for iodine sorption determines their low CT values over the whole staining process. Therefore, longer immersion periods with a low concentration of iodine only improve their CT contrast in a limited way. In terms of the acquisition of CT contrast for the whole specimen, the connective tissues are not a big issue because they do not block the diffusion of iodine. The lower CT values of connective tissues allow them to be adequately distinguished from the surrounding tissues with much higher CT values (like muscles and epithelia), although they may make effectively distinguishing different layers within connective tissues challenging. Another factor influencing staining is the bone. We have observed at most a very limited increase in CT values for bone over the staining process using low-concentration iodine and report that well-ossified adult bone may block diffusion of contrasting agent into the bird brain (Fig. 4). A much more concentrated staining solution could potentially enhance diffusion through bone because of the increased concentration gradient; this suggestion should be adopted with caution while considering the serious shrinkage effect caused by more highly concentrated iodine solutions [6, 9]. We also suggest, for staining tissues isolated by or encapsulated by bony structures, that injection of contrasting agent directly through bones to the target tissues is an option.

It should be noted that no substantive shrinkage (<1 %) was observed in the bony tissue by the linear measurements of same structures between different scans in our experiments. Soft-tissues (e.g., muscles) showed only minor shrinkage; less than 5 % in linear measurements. These muscle shrinkage values are much smaller than the up to 70 % change in volume observed in mouse hearts when a much higher concentration I_2_KI (as Lugol’s iodine) was used with a shorter staining duration [12]. The shrinkage observed here is comparable to that reported from the staining of a whole-mounted embryonic specimen using a similar concentration of iodine (as Lugol's iodine) to the 2.97 % iodine-based neutral buffered formalin solution used here [6].

### Integrated interpretation of the staining mechanism from the experimental data and modeling

Our modeling suggests that the increase in the partition coefficient (*K*_*d*_) leads to an increase in the retardation factor (*R*_*t*_) and further decreases the effective diffusion coefficient (*D/R*_*t*_). This result reveals the passive diffusion limit of iodine observed in muscles and other tissues with a high partition coefficient (*K*_*d*_) and a high bulk density (*ρ*_*b*_). The finding also explains why a long staining period was effective for these tissues. Our results suggest that the increase of the constant flux at boundary condition (Fig. 7:  x  = 0) would raise the peak value of the iodine concentration at the boundaries (Fig. 6: Bound 1 and Bound 2). This finding indicates that solution with higher concentration can lead to higher saturated iodine values in exterior tissues but may not be effective for the interior tissues.

The accumulation of iodine near the boundary between connective tissues and muscular tissues was primarily due to the difference in sorption capacity between the two interior zones; i.e. the higher *K*_*d*_ in muscles compared to the lower *K*_*d*_ in connective tissues. Based on the staining results, the retardation factor *R*_*t*_ (=1+ *ρ*_*b*_*K*_*d*_*/θ*) was much smaller in connective tissues (*ρ*_*b*_-bulk density, *K*_*d*_-partition coefficient, *θ* -porosity; see Methods for detail definition). Therefore, the effective diffusion coefficient (*D/R*_*t*_) is greater in connective tissues than in muscular tissues [5]. Iodide molecules diffused at a moderate rate through the skin, and much faster in the connective tissues, and dramatically slowed down at the edge of muscle due to the binding of iodine with glycogen in the muscles. The differences in transport rates and binding ability between tissues led to the accumulation of iodine at the outer boundary of muscular tissues (Fig. 6: Bound 2) as our modeling and experiments show. According to the equation for the retardation factor, tissues with higher porosity (*θ*) and lower bulk density (*ρ*_*b*_) tend to have a higher effective diffusion coefficient; and therefore, if other conditions are all similar, the staining rate observed among different tissues of similar composition can be explained by their structure differences (e.g., porosity and bulk density). Faster staining occurred within tissues that have a higher porosity and a lower bulk density and may explain why the lens and epithelium stained faster than muscles in our experiments. It is instructive to compare the iodine concentration increase between Model d15 to d0 and Model d30 to d15 (Fig. 7). The binding of iodine first with the exterior tissues prevents a similar contrast increase within deeper interior tissues. As the exterior tissues were approaching or reached a saturated state for iodine binding, larger increases in CT contrast and in iodine concentrations are seen in the more interior tissues. This pattern provides a theoretical and quantitative basis for the difficulty observed in staining “larger” specimens.

The lack of effective staining increase between scan A and scan B could be explained by the lack of sustained flux at the boundary. The increase of the solution concentration at C_0_ (initial concentration) would significantly increase the staining effect or the iodine concentration in the skin, at muscle boundaries, and the region sufficiently close to the boundary. It would not have a big effect on interior tissues during early staining. This finding has practical importance considering the over-staining effect of muscle tissues reported in smaller specimens [6, 9]. Therefore, for staining larger samples, we propose that the optimal strategy would be a combination of using an initial low-concentration iodine solution, with regular replacement with a slightly higher concentrated solution, and the adoption of a long duration to alleviate the “unequal staining effect”. This “unequal” refers to the coexistence of the over-staining in the exterior muscle regions with insufficient staining in the more interior regions [9].

The investigation of the D-S model provides a first attempt to explore the staining mechanism through comparative assessment of serial CT results with appropriate numerical modeling. The reaction of iodine with tissues (i.e., sorption) is an important factor to be considered when estimating the effective diffusion rate in different samples. Through our simulations, we have demonstrated that this interaction could dramatically slow down the effective diffusion rate of iodine and delay the arrival of iodine at more interior regions in tissues. This effect is shown to be especially important in those tissues (e.g., muscles), which have a large partition coefficient (*K*_*d*_), high bulk density (*ρ*_*b*_), and low porosity (*θ*). Our conclusions are consistent with previous observations of the high binding capacity of iodine with muscles [14, 16]. The reaction of glycogen with iodine appears to be a less serious issue for the staining of epithelium as shown in the CT value plots (Figs. 1, 2). This variation could be a result of different structural properties of the epithelia relative to those of muscles although their partition coefficient (*K*_*d*_) are equally high. For connective tissues, their lower partition coefficient determines their lower CT contrast values compared to other tissues (e.g., epithelia, muscles), even when using long staining durations.

### Proposed staining mechanism at the molecular level

The mode of iodine staining at the molecular scale remains poorly understood [5]. The general previously-proposed explanation involves the sorption and/or binding of the iodine with the most common components of animal cells (e.g., glycogen, lipids) and creation of resultant glycogen-iodine or lipid-iodine complexes [14, 16]. Due to the limited scope of this study, our simulations do not take the rate of this reaction or other aspects of chemical kinetics into consideration.

Previous spectral analyses of different iodine complexes (e.g., with amylose, amylopectin, and glycogen) suggested that the potential chemisorption is related to the formation of polyiodide units (e.g., [I_5_]^−^, [I_7_] ^-^) with the linear or branched carbohydrate [19]. The sites where these iodine molecules would be bound in these polymers are the exterior “A-chain” and the interior “B-chain” [20]. The formation of a helical structure is important to stabilize the polyiodine in amylose-iodine (AI), amylopectin-iodine (API), and very likely to the Glycogen-Iodine (GI) complex [20–22]. The complex behavior of the glycogen-iodine composition might be related to the involvement of both the “A-” and “B-chain” for binding iodine in glycogen molecules. The differences in average chain length between glycogen and amylopectin have also been used to explain their different spectral absorptions and different binding properties with iodine [21]. Previous experiments have suggested that the shorter helices of the GI complex in comparison to AI can explain their lower stability [14]. In addition, the interior “B-chain” with its multiple branches may be less optimal for building the rigid helix for the stabilized long polyiodide chains [19]. Instability of the GI complex may thus explain the difficulty in staining tissues here and in previous experiments especially when a low concentration of iodine solution was used.

Increased staining effects observed with the continued input of new iodine into the system suggest that the higher concentration of the staining solution during equilibrium may facilitate the stable binding of iodine within the helical glycogen complex in tissues. The progressive increase in bound iodine could relate to a step-wise pattern in the formation of polyiodine with both the “A-” and “B-chain” in glycogen, especially given their sequential binding; the “A-chain” is suggested to bind first in AI and API complexes [20].

### A complex pattern of change in iodine concentrations in the staining solution

**Fig. 8:**
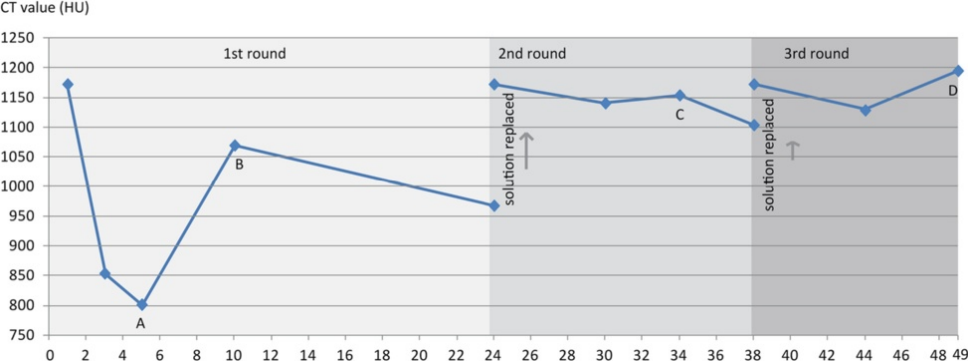
The changes in CT values of the staining solution over time. The trend line represents an estimation of the changed iodine concentration in solution throughout the days of staining. Blue dots show the scanned staining solution extracted during the experiment. **a**, **b**, **c**, and **d** were labeled as the indication (scan A, B, C and D) of when the sample were scanned during the staining

In addition to scans of the sample, we also scanned the solution extracted during the staining process (9 samples in total). These data revealed a complex pattern with respect to the change of iodine concentration in the staining solution (Fig. 8). A repeated pattern in the iodine concentration in the staining solution was discerned over the three rounds of staining although the amplitude of the change differed significantly. From the initial iodine concentration (around 1173 HU for CT value; equal to 2.97 % w/v for concentration), there was a significant drop of CT values in the solution for the first 3–6 days of staining consistent with previous results in which the best staining effect occurred in the earlier staining periods [9]. Following this initial period desorption appears to have occurred as indicated by the rebound of iodine concentration in remaining solution (Fig. 8). This desorption period may be due to a shift in the differential concentration of iodine in the solution and within the specimen. The fall-and-rise pattern seen was repeated during day 1 to 10, day 24 to 34, and day 38 to 49, but the amplitude of latter two cycles was much smaller than that observed in the first.

The diffusion-relaxation sorption behavior found in synthetic polymer-solute systems might explain the interaction of iodine with the organic tissues (e.g., epidermis, connective tissues, epithelium, and muscles). A diffusion-relaxation model was proposed to explain anomalies in the interaction of various solutes and polymers [23]. Anomalous diffusion was summarized as an initial maximum uptake, followed by temporary desorption and subsequent resorption in a system [24]. If we consider the whole goose head as millions of polymer aggregations, then the pattern in the staining solution concentration discerned here could be similarly explained by a diffusion-relaxation model.

### Conclusion

**Fig. 9:**
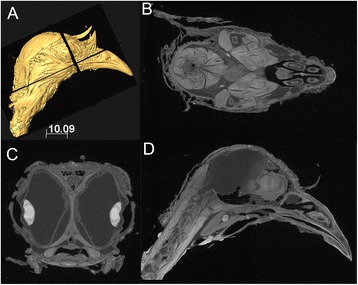
CT images and 3D rendering of a large specimen (a tinamou head) scanned using the staining approach proposed (see text for detail). **a**: 3D rendering of the specimen; **b**: coronal section; **c**: horizontal section; **d**: sagittal section. The positions of where these sections were taken are shown as plan insets (**a**). Scale unit in **a**: mm

Our experimental results should be useful to other researchers in developing their own appropriate staining protocols (e.g., staining duration, concentration, and specific enhancement) for staining large samples with different tissue types. The passive diffusion limit of iodine in tissues was implicated for the lack of effective staining of the interior regions of large samples [5, 9]. Here, we show effective staining of these interior regions of such sample can be achieved through a progressive staining approach (Figs. 1, 2, 3, 4 and 5), although this approach requires a long staining duration. This progressive staining approach using iodine solution starts with a relatively low concentration previously reported to be effective in the clinical diagnosis of carcinomatous tissues within human tongue [25]. A step-wise increase in the concentration of the staining solution during solution replacement (e.g., 1 %, 2 %, and 3 %, respectively) is further suggested here. The steadily increased iodine concentration would maintain a certain concentration difference between solutions and specimen during the whole process. Such an approach has yielded ideal results on another sample treated, a tinamou head (Fig. 9). This strategy potentially avoids over-staining effects for exterior regions of the sample and also seems to avoid the dramatic shrinkage reported when using a high concentration iodine solution at the beginning of staining [12]. As shown in both our experimental results and in simulation, unequal staining effects for large muscles are a result of staining inefficiency over the short staining durations investigated previously. Using serial solution replacement and longer staining durations with low concentrations, this previously identified issue has been resolved; the approach taken here yields almost equally stained exterior and interior muscle regions for large muscles (Fig. 9).

The sorption of iodine by tissues affects the staining process in two ways. On the one hand, it could dramatically increase contrast through large sorption in tissues with high partition coefficient. This is clearly seen in the staining of muscle versus staining of connective tissues. On the other hand, it could also significantly retard the effective diffusion of iodine into interior tissues and dramatically slow down the staining of these tissues. As a result, for passive diffusion, a continuous incubation over a few weeks, or even months, with the progressive input of iodine is required for these muscles, as shown in our experimental results. For tissues with a low partition coefficient, simply lengthening the immersion period would not significantly increase their CT values. But given the arrangement of connective tissues (e.g., fasciae) within organic specimens, the lower CT values are usually not a problem for the acquisitions of soft-tissue contrast as a whole. The increase of attenuation in soft-tissues by the sorption of the contrasting agent would reduce their distinction from bone in CT scans because well-mineralized tissues do not stain at the same rate. The need for maintenance of a distinction between soft-tissues and bone needs to be weighed against the efficiency of rapid, high-concentration staining. Choices regarding staining duration and concentration should be made based on target tissue types and size. For specimens with a number of distinct target tissue types, the relative organization of these tissues (e.g., in the large viscera or the cranium) should also be considered.

In addition to diffusion limits, another limiting factor in long staining procedures may be maintenance of the stable GI complex. Because we do not have adequate data on the reaction rate for the formation of GI complex, how long effective staining would last remains unknown. Over the earlier staining periods (e.g., scans N-A-B), stagnation of the staining effect may be an indication of the sorption rate of GI complex is approximately equal to the rate of its decomposition or degradation. The low binding energy of iodine (IBE) explains the low stability of GI complex in comparison with that of other iodine-polymer compounds [14]. Therefore, another factor in the effective staining of large samples is related to countering the effect of the degradation of the GI complex over time.

## Methods

### Specimen processing and staining protocols

An adult Greater White-fronted Goose (*Anser albifrons gambelli*) was salvaged from west Texas near Egypt in the fall of 2013. The donor’s Texas hunting License is 348160038189; the specimen was accepted by the University of Texas, Vertebrate Paleontology Laboratory under the Federal Bird Permit MB52556B-0, Special Purpose Possession (Dead Migratory Birds for Educational Use, With Salvage) and Texas state permit (Educational Display Permit) EDU-1213-179.

The salvaged specimen was processed at the Texas Natural History Collections facility (TNHC). It was decapitated between cervicals three and four, and then fixed in 10 % Neutral Buffered Formalin (NBF) for two weeks before being transferred into an enclosed jar containing 600 mL 2.97 % (w/v) iodine-based buffered formalin solution for staining. The use of formalin as a small part of the solvent rather than exclusively using water was preferred to prevent potential tissue deterioration given the long staining period. The solution was prepared by adding 6 g iodine (Iodine, ACS reagent, ≥99.8 %), 12 g potassium iodide (Potassium Iodide CERT ACS) into 600 mL Neutral Buffered Formalin (10 %) solution. The actual concentration of this iodine-based solution is slightly smaller than 3 % (i.e., 2.97 %) due to the volume increase after the mixture of new solutes (I_2_, KI). In addition, the calculation the precise iodine concentration would not be affected by using buffered formalin as the solvent because no evidence has indicated the binding of iodine with formaldehyde or sodium phosphates contained in the solution. Even using the Lugol’s iodine for staining, some formalin residue will also be present from the prior fixation. The volume of the goose head sample (head and the small portion of the attached neck) is approximately 125 mL. The choice of a low iodine concentration was influenced by previous experiments in which muscle tissues would approach the X-ray attenuation level of bone in CT images when higher iodine concentrations were used [26]. Low concentrations were also preferred to reduce the possibility of tissue shrinkage observed at higher concentrations (e.g., 20 % w/v; [12]); lower concentrations (e.g., 3.75 % w/v I_2_KI) have been proposed to minimize shrinkage [6]. Shrinkage more generally has been explained by the movement of water from tissues to the staining solution due to establishment of an osmotic gradient [6, 15].

After formalin fixation, a CT scan was acquired to function as a control (no-stain or “scan N”; Figs. 1, 2, 3, 4 and 5, N) before the staining started. Staining began on day 1 and the specimen was scanned on day 5, 10, 34, and 49. These scans are referred to as scans A, B, C, and D, respectively. The staining solution was replaced (600 mL) by new iodine-based buffered formalin solution prepared in the same way (2.97 %, w/v concentration) on day 24 and day 38. The replacement of iodine maintains the concentration difference between the solution and the specimen, which made the diffusion sustainable in the long run. This process is further investigated through modeling.

### Scanning parameters, beam hardening, and artifacts

Scanning was done using a custom instrument built by North Star Imaging (Rogers, MN) and operated at the University of Texas High-Resolution X-ray CT facility. X-ray parameters (voltage, current, and power) were the same for all five scans (150 kv, 0.24 mA); slight differences in the source-object distance (<7.9 mm) resulted from minor orientation differences for the specimen among the specimen scans (N, A, B, C, and D), and a scanner software update between scans B and C that changed some instrument parameters. The specimen was tightly wrapped with plastic and scanned in air. Data acquisition was done in a helical cone-beam mode [27], in which the specimen was raised continuously with respect to the beam during rotation. This method enabled efficient scanning of “tall” specimens and avoids cone-beam artifacts that can occur on horizontal surfaces near the top and bottom of the cone field. Use of the same parameters for each scan was intended to allow CT values measurements for the same tissue at a same place within the sample to be interpreted as representing differences due only to the staining effect. However, the scan images included beam-hardening artifacts that could not be fully removed with corrections provided by the scanning software. This was in part a consequence of the large sample size and the large amount of iodine in the image field, which increased the severity of beam-hardening artifacts. In addition, the images also show a spiral “barber pole” artifact, a field of slight brightening stemming from the helical acquisition and reconstruction [28].

We applied a post-calibration correction to the raw CT values individually for each data set to correct for beam-hardening and helical artifacts. This correction involved comparing the CT values of the air encapsulated in the nasal cavity and of the tissue in the center of the brain for the five scans. Grayscale values for the same material (the unstained brain and the air) are expected to be the same in different scans using the same acquisition parameters; however, we observed minor variations in these materials (Table 1). The encapsulated air and the unstained brain tissue were selected to conduct the recalibration because they showed fewer beam hardening artifacts across scans. Both air and brain tissues were used for the recalibrations, by which the grayscale of these two materials was rescaled as the same mean values for scans of N, A, and B, and separately for scans of C and D (Table 2). Separate calibrations for the earlier and latter scans was based on consideration of the voxel size, which was more similar among earlier three scans (0.0858 mm, 0.0858 mm, and 0.081 mm for N, A, and B) and in the later two scans (0.0757 mm and 0.072 mm for C and D) respectively due to scaling in the reconstruction algorithm and the previously mentioned software update.

**Table 1:** Inter-calibration of pixel intensity (grayscales) to account for scanning artifacts detected (see Methods); X represents the raw measurements for grayscales and the calibration equation was applied to correct scanning artifacts.

Scan	Timing	Air	Brain	Range	Average air	Average range: brain-air	Calibration equation
N (control)		11988	21532	9543	13192	8635	X *8635/9543 + 13192-11988* 8635/9543
A	Day 5	13415	21113	7699	(N, A, and B)		X *8635/7699 + 13192-13415* 8635/7699
B	Day 10	14174	22835	8661			X *8635/8661 + 13192-14174* 8635/8661
C	Day 34	14335	23019	8685	15206	8278	X *8278/8685 + 15206-14335* 8278/8685
D	Day 49	16078	23950	7872	(C and D)		X *8278/7872 + 15206-16078* 8278/7872

* represent multiple; original measurements and calculation were conducted with two decimals and rounded to integer shown here

**Table 2:** Correlation of grayscales, CT values (Hounsfield Units), and iodine concentration in scans of the staining solution. The Hounsfield Units of I_2_KI solution (in italics) was calculated through a lineal regression between the Grayscales values and Hounsfield Unit of aqueous formalin solution and air (see Fig. 10a)

Materials	Grayscale values	Hounsfield unit	Iodine concentration	
			mmol/L	w/v
Aqueous buffered formalin solution	18202	0	0	0
I_2_KI-buffered formalin solution	21206	*394*	66	1 %
I_2_KI-buffered formalin solution	24552	*949*	199	3 %
I_2_KI-buffered formalin solution	28058	*1531*	398	6 %
I_2_KI-buffered formalin solution	31978	*2181*	583	8.8 %
Air	12800	−1000	N/A	N/A

### Quantitative comparison of iodine concentrations in solution and tissues

**Fig. 10:**
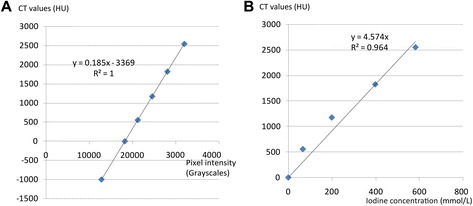
The relationship between CT image intensity (grayscales) with CT values (Hounsfield Units; **a**), as well as with estimated iodine concentrations (**b**). Also see Table 2 for the data points used

To estimate the concentration of iodine within cranial tissues, we first assessed the iodine within the solution with a separate scan of a small vial containing only the buffered formalin solution, and four vials of I_2_KI buffered formalin solution of known concentrations (i. e., 1 %, 3 %, 6 %, 8.8 % w/v; Table 3). The 8.8 % I_2_KI solution were made by adding 3 g I_2_ and 6 g KI to 100 mL formalin solution. An ideal mixture of solutes to the solvents was assumed. Then this solution was diluted into 6 %, 3 %, 1 % by adding the whole solution up to 150 mL, 300 mL, and 900 mL with added buffered formalin solution subsequently. Only a small vial (around 1 mL) of these four concentrated solutions (1 %, 3 %, 6 %, 8.8 %) were extracted during the preparation. The effective change of concentration due to this extraction was very minimal and ignored here. In the Hounsfield scale [29], the CT values of air and water (close to the buffered formalin solution here) were defined as −1000 and 0 Hounsfield Units (HU) respectively. Based on CT values of these two materials (air and water), all measured grayscales (Table 2, Fig. 10a) were converted to the equivalent CT values (in Hounsfield Units) along a linear regression following the previous approach [29]. A linear relationship between iodine concentrations (mmol/L) was correlated with the CT values (HU) and its slope was described by a calibration factor (i.e., ‘K’ factor, [29]). The value of ‘K’ factor can vary in different scanning systems [29]. The ‘K’ factor is 4.6 (HU/mmol) in our regression (Fig. 10b); it was calculated to be 3.9 (HU/mmol) in a previous study (for I_2_KI solution, [6]). The conversion of grayscale values to CT values (Hounsfield Units) here served to facilitate comparison among scans in this study and also to facilitate the comparison of staining effects with those from previous studies [1, 2, 4, 6].

**Table 3:** Diffusion parameter assumption (*θ*, *ρ*_*b*_) and calibrated results (*D*, Flux, *K*_*d*_) in the Diffusion-Sorption modeling

Parameters and units	Zone 1 (skin)	Zone 2 (connective tissue)	Zone 3 (muscle issue)
Range (mm)	0-0.7	0.7-0.8	0.8-15
*θ* (porosity)	0.8	0.8	0.8
*ρ*_*b*_ (kg/L)	0.16	0.16	0.265
*K*_*d*_ (L/kg)	200	100	200
Initial concentration (mmol/L)	0	0	0
*D* (m^2^/s)	1*10^−9^	1*10^−9^	1*10^−9^
Flux (*N*_*bc*_) (m*mmol/(L*s))	1.4*10^−6^		

*Notes*: *ρ*_*b*_ is the bulk density of different tissues; it was calculated as the mass of the dry tissue divided by total volume. For instance, 1 unit of muscle (i.e., one liter) has a mass of 1.065 kg [31] with dry muscle’s mass 0.265 kg; then *ρ*_*b*_ = 0.265 kg/L

* represent multiple

### Diffusion-Sorption (D-S) model

We used a one-dimensional Diffusion-Sorption (D-S) model with a linear sorption isotherm to simulate the solute transport through heterogeneous domains [17, 18]. The D-S model was developed and is commonly used for, simulation and prediction of solute (e.g., contaminant) migration for domains in which reaction occurs along transport pathways in porous media (Fig. 6). The diffusion of iodine within tissues is a complex and complicated system that has not been fully understood theoretically [22]. The simulation explored here was intended to assess possible fit with observed values from the imaging and staining process and to explore how much tissue differentiation (e.g., duration and propagation) may be explained by the simple process modeled. In this study, the transport of iodine is described by the following equation:

1 $$ \left(\theta +{\rho}_b\ast {K}_d\right)\frac{\partial c}{\partial t}=\theta \ast D\frac{\partial^2c}{\partial {x}^2} $$

where θ is porosity (0–1), ρ_b_ is bulk density (kg/L), *D* is diffusion coefficient (m^2^/s), and the partition coefficient *K*_*d*_ describes the sorption of iodine between solid phase (tissue) and solute phase (i.e. *K*_*d*_ = *c*_*solid*_/*c*). The iodine concentration in the solid tissue (*c*_*solid*_) is measured as a ratio, which is the weight of iodine to the weight of solid tissue (w/w); the iodine concentration of solute (*c*) is measured as the weight of iodine to the volume of solvent, with a unit of kg/L. Therefore, the unit of *K*_*d*_ becomes L/kg. Porosity is a parameter that determines the degree to which the infiltration of solution can easily occur within tissues; the defined porosity [17] is the void volume per unit volume of porous media. Here, the “analogous porosity concept” applied for organic tissue is the vascular portion of a specific tissue. The bulk density *ρ*_*b*_ (kg/L) references the density of dry tissue (without water), and was calculated by the weight of dry tissue (net weight after water removed) divided by the total volume of tissue.

The retardation factor, *R*_*t*_, was previously defined as (1+ *ρ*_*b*_*K*_*d*_*/θ*) [17], and it represents the ratio of pore-water velocity to the solution (iodine solution) velocity. Then Eqn 1 is simplified to

2 $$ \frac{\partial c}{\partial t}=\frac{D}{R_t}\ast \frac{\partial^2c}{\partial {x}^2} $$

Here, *D*/*R*_*t*_ represents the effective diffusion coefficient. It equals the diffusion coefficient of a chemical under non-adsorbing conditions divided by a retardation factor of the adsorbing system [17]. In this study, the effective diffusion coefficient is an important factor expected to affect staining rate in the experiments. For the staining effect, we are interested in the total concentration of iodine within the porous media, which is the total iodine concentration in the tissues and in the pore fluid; and it can be represented by

3 $$ {C}_T=\left(\theta +{\rho}_b{K}_d\right)\ast c $$

In our simple simulation, the diffusion domain had three homogeneous zones, a narrow exterior zone (e.g., epidermis, and dermis) which was directly in contact with the staining solution, and another much wider internal “median muscular zone”. Between the exterior (e.g., epidermis and dermis) and the internal muscular zone, a very thin-layered connective tissue zone was defined (for the fascia). Given the extreme complexities of tissue types in the real condition within the whole head, we restricted our modeling to only one dimension and excluded other tissue types such as bone and cerebrum. For this reason, only the tissue in the linear transects chosen (e.g., through the adductor chamber) was assessed (Fig. 3e: *b*-*d*). The three zones defined here each had their own partition coefficient (*K*_*d*_). The partition coefficient is an intrinsic attribute for a specific tissue and is calibrated through the modeling process. Larger *K*_*d*_ indicates more iodine would be held within the tissues during equilibrium and suggests a higher capacity for iodine sorption for a tissue.

The total concentration of iodine in the skin, connective tissue, and muscular tissues increased with time due to the diffusion of iodine from staining solution to the study region. To further simplify the model for the simulation, we assumed that there is a constant flux (*N*_*bc*_) boundary condition at *x* = 0, which needs to be calibrated. Because diffusion of iodine was limited within approximate 2 cm from the boundary of *x* = 0, domain was treated as semi-infinite [30]. The domain for the simulation was set to 0.03 m in length, and a non-flux boundary condition was specified at *x* = 0.03 m. Initial concentration *c* was zero in the model. The simulation under the D-S model was implemented using COMSOL Multiphysics, and four parameters, i.e. three partition coefficient (*K*_*d*_) for the three zones (skin, connective tissue, and muscle), and flux *N*_*bc*_, were calibrated by fitting the model to experimental measurements (Table 3).

### Analysis of experimental data

Because of the minor orientation discrepancy among different scans due to manual positioning of the sample, resampling of the 3D datasets was conducted in Avizo 8.1 (FEI Visualization Sciences Group) to optimize the congruence of the regions studied in detail (Figs. 1, 2, 3, 4 and 5). Reslicing of the specimen at similar positions was accomplished in Avizo 8.1 by choosing the same three rigid anatomical points and fitting them with a plane in each of the different scans. By measuring CT values (in HU) at approximately the same sections of the five scans in nearly identical positions, a series of the grayscale measurements were obtained in “ImageJ v1.48” in the 16bit-tiff images (grayscale value range 0-65535; Figs. 1, 2, 3, 4 and 5; the straight yellow line). The sections were selected to include different tissue types. Pixel intensities were converted to CT values as described above. The adjusted values (to correct for beam hardening and other artifacts described above; see Table 1) were used to plot CT value profiles in target regions of interest in each scan (Figs. 1, 2, 3, 4 and 5). The occurrences of peaks and valleys along the X axis (Figs. 1, 2, 3, 4 and 5) were identified as the major changes of tissue type and were aligned with a similar position (e.g., Fig. 3e: *a*-*d*). These steps ensured that the comparison of staining effect was made on the same tissues at the same position over the experimental time period. The minor discordance of these curves was caused by the slight change in the position of the soft-tissue among scans due to the Avizo-reslicing and interpolation not being perfect.

To gather experimental data for the simulation, we chose a line in the cross section of the adductor chamber (Fig. 3). The tissue types represented by the selected line are simple along the major proposed diffusion direction (Fig. 3e: *b* to *d*), and included the exterior zone, the interior muscles, and a very thin connective tissue in between. The line was also long enough (around 1.5 cm) to demonstrate the proposed diffusion progress. The relatively long incubation period (over 5–10 days) for each round of staining enabled the achievement of equilibrium for both the micro-scale reaction (i.e., iodine bound) and macro-scale diffusion within tissues based on previous experiments ([6], Li, pers. obs.). Equilibrium was assumed to be reached as no further exchange was occurring between the staining solution and tissues.

The CT values for the measured tissues were further converted to equivalent iodine concentration (Table 2). For the fixed muscles, the converted concentration was approximately 150 mmol/L in the non-stain scan (N), and this offset was subtracted from the measurements of all scans (N, A, B, C, and D). When comparing the transformed concentration of scan A and B with the non-stain scan (N), a higher CT value (Fig. 3) and converted iodine concentration in the interior muscle was observed although the region was clearly not stained in these treated specimens. This increase in CT values for the non-stained tissues of the treated specimens could be the result of a fast infiltration of solute (i.e., lighter potassium ion) that slightly increased the density without iodine; iodine (or iodides) took longer to infiltrate because it was rate-limited by the sorption process. However, it also might be an indication that the method to identify and remedy grayscale variation due to image artifacts was not perfect in all cases. Another correction was made (minus 50 mmol/L) to remove this offset in scans of A, B, C, and D.

### Availability of data and materials

The de-stained goose specimen (TMM M-14998) is deposited at University of Texas at Austin, Vertebrate Paleontology Laboratory. The CT imaging serial dataset will be available on ‘digimorph.org’ and will also be deposited in the Dryad repository.

## Abbreviations

AI: amylose-iodine
API: amylopectin-iodine
*c*: concentration
C_0_: initial concentration of the staining solution
*D*: diffusion coefficient
D-S: Diffusion-Sorption
GI: Glycogen-Iodine
HU: Hounsfield Units
IBE: binding energy of iodine
I_3_^−^: triiodide
*K*_*d*_: partition coefficient
micro-CT: micro-computed tomography
*N*_*bc*_: boundary flux
*R*_*t*_: retardation factor
*ρ*_*b*_: bulk density
*θ*: porosity
3D: three-dimensional
